# The Importance of the Nurse Cells and Regulatory Cells in the Control of T Lymphocyte Responses

**DOI:** 10.1155/2013/352414

**Published:** 2012-12-26

**Authors:** María Guadalupe Reyes García, Fernando García Tamayo

**Affiliations:** Laboratorio de Inmunobiología, Departamento de Biología, Facultad de Química, Universidad Nacional Autónoma de México (UNAM), Ciudad Universitaria, 04510 México, DF, Mexico

## Abstract

T lymphocytes from the immune system are bone marrow-derived cells whose development and activities are carefully supervised by two sets of accessory cells. In the thymus, the immature young T lymphocytes are engulfed by epithelial “nurse cells” and retained in vacuoles, where most of them (95%) are negatively selected and removed when they have an incomplete development or express high affinity autoreactive receptors. The mature T lymphocytes that survive to this selection process leave the thymus and are controlled in the periphery by another subpopulation of accessory cells called “regulatory cells,” which reduce any excessive immune response and the risk of collateral injuries to healthy tissues. By different times and procedures, nurse cells and regulatory cells control both the development and the functions of T lymphocyte subpopulations. Disorders in the T lymphocytes development and migration have been observed in some parasitic diseases, which disrupt the thymic microenvironment of nurse cells. In other cases, parasites stimulate rather than depress the functions of regulatory T cells decreasing T-mediated host damages. This paper is a short review regarding some features of these accessory cells and their main interactions with T immature and mature lymphocytes. The modulatory role that neurotransmitters and hormones play in these interactions is also revised.

## 1. Background

Lymphocytes are cells that express receptors that can recognize foreign antigens and activate inflammatory reactions in their surroundings to eliminate them. In this way, lymphocytes provide specific adaptive immunity in all vertebrates. These cells are classified into two main subpopulations, B and T, which recirculate inside the peripheral blood and lymph vessels distributed through the entire body, but lymphocytes can also migrate across the high endothelial cells of venous capillaries and home to different organs where foreign antigens are located. When the lymphocyte's receptors recognize them, the lymphoid cells proliferate and form clones that start a wide set of specific defensive humoral and cellular responses to eliminate microorganisms and infected or malignant cells [1]. In this paper we will refer only to T lymphocytes.

The development of T lymphocytes in the thymus and the control of their functions in the periphery are mainly controlled by two special cell populations named “nurse cells” and T regulatory cells. Early in their development, immature T lymphocyte precursors migrate inside the thymus gland and are engulfed by epithelial “thymic nurse cells” (TNCs) [2], which stimulate their development and simultaneously remove most of the defective or self-reactive T lymphocytes. Once the remaining mature T cells leave the thymus, they are subjected to a second control by “regulatory cells” that can inhibit their excessive or dangerous responses [3].

The thymic nurse cells eliminate within their vacuoles high affinity autoreactive and poorly developed T lymphocytes, thus preventing subsequent autoimmune reactions and diseases. The peripheral regulatory cells instead, only reduce the functions of circulating T lymphocytes. While the nursing work kills and negatively selects defective T lymphocytes [4], the regulatory work only suppresses T lymphocyte-mediated inflammatory reactions, supporting the immune tolerance and dampening hypersensitivity responses [5]. The normal modulator activity of nurse cells and regulatory cells can be also disrupted in the course of diverse chronic infectious diseases, particularly those with a parasitic etiology.

### 1.1. T Lymphocytes Migration

T lymphocyte progenitors enter the thymus via the bloodstream by using integrins, selectins and chemokines during periodically receptive times that are spatially and temporally regulated. Once intrathymic niches are saturated with T cell progenitors, no other new T cell progenitors are allowed to enter until the former move on and leave the niches empty [6]. T cell development in the thymus is more active from fetal to perinatal stages and declines with aging [7].

The intrathymic route of immature T lymphocytes and their eventual development involve an ordered and regulated movement of their progenitors that follow chemokine gradients and interact with adhesion molecules such as integrins, P-selectin, neuropilin-1, and semaphorin-3A [8] through thymic cell networks until they are in the care of nurse cells [9].

Acquiring functional T cell receptors and coreceptors and recognizing self or non-self antigens are the first decisive steps in the life of immature T lymphocytes in the thymus. In the course of their intrathymic migration, T lymphocyte precursors engage their bidirectional interactions with TNCs. Initially they are named double negative (DN) cells, since they do not express neither the CD4 nor CD8 coreceptors. The development of these immature lymphocytes consists of four stages (DN1, DN2, DN3, and DN4) according to the expression of the CD4 and CD8 coreceptors [10] on the membrane surface. As the migration goes on, T lymphocytes begin the expression of their receptors to recognize antigens called T cell receptors (TCR).

In the DN3 stage of their development, immature T lymphocytes begin the expression of a pre-T cell receptor. Signaling pathways through the intracellular domain of the Notch1 transmembrane protein [11] and others [12] control the recombination and rearrangement of the V(D)J gene segments of the *α*, *β* or *γ*, *δ* chains of these pre-TCRs. Furthermore, the expression of receptors to recognize antigens in immature T cells is influenced by endogenous cytokines [13], which also modulate the migration of immature lymphocytes through thymic epithelial cells and their subsequent selection.

Once their maturation begins, T lymphocytes produce interferon gamma (INF-*γ*), which controls the expression of the fibronectin and laminin receptors on the thymic epithelial cells (TECs), modulates thymocyte adhesion to thymic epithelial cells, induces the expression of the human leukocyte antigen DR in human thymic epithelial cells, and finally is also involved in thymocyte selection and their subsequent release from the TNC vacuoles [14].

Lymphocyte stimulation by TNC promotes the expression of T cell receptors to recognize antigens and CD4 and CD8 coreceptors even though lymphocytes have not yet completed their development. Maturing lymphocytes must pass through an intrathymic selective process to avoid apoptosis. Lymphocytes stay alive and leave the thymus only after proving that their receptors do not recognize self-antigens with high affinity. To this purpose, the anatomical integrity of the thymus and the efficient function of their nurse cells to perform the first selective and dangerous event in the life of T lymphocytes are necessary.

Different exogenous molecules including hormones such as oxytocin, neurotensin, insulin growth factor 2 (IGF-2), vasopressin [15], glucocorticoids [16], androgens [17], and estrogens [18] and neurotransmitters produced by the autonomic nervous system [19] such as acetylcholine [20], histamine, and serotonin [21] also influence the migration of T lymphocytes by promoting the expression of adhesive molecules.

### 1.2. Thymic Involution

The competence of the immune system depends on thymic abilities to support developing T lymphocytes and eliminate high affinity autoreactive cells. However, thymic activities do not remain constant throughout life. In fact, the size and cellularity of the thymus undergo a physiological decline almost immediately after birth. In adolescence, when the production of sexual hormones increases, thymic involution accelerates and the number of thymocytes gradually decreases at a rate of 3–5% per year; in adulthood, the number of thymocytes continues its decline at a rate of 1% per year [22]. Furthermore, transitory increases in the production of steroid hormones reduce thymic cellularity during pregnancy [23], and the thymus reduces both its volume and thymocyte numbers [24] during chronic stress. These changes influence the functions of nurse cells and regulatory cells, thus affecting the number and competence of different T lymphocyte subpopulations that flow into the peripheral circulation.

Studies on thymus-hormone interactions have shown that thymic involution and T lymphocyte deficiency of aged male rats could be reversed by reducing testosterone levels through surgical castration [25] or by administering the antiestrogenic agent tamoxifen [26]. The elevation in sex hormone levels is associated with the natural involution of the thymus gland since testosterone induces the apoptosis of CD4^+^CD8^+^ double-positive developing thymocytes [27] via the tumor necrosis factor-alpha (TNF-*α*).

Besides, despite the relation between pregnancy and thymic involution [28], other authors [29] have shown that elevated progesterone levels can increase the number and functions of peripheral thymus-derived T cells. Pregnancy and estrogen treatments improve Treg cell functions and enhance the expression of their Forkhead-winged helix box p transcription factor 3 (Foxp3) [30]; other authors [31] have reported similar effects after using glucocorticoids in asthmatic patients. Results suggest that hormones and other factors do not always reduce the T lymphocyte number and functions. Although apoptosis decreases the T cell activities during aging [32], thymic involution is also associated with an increase in Treg cell numbers in peripheral blood [33, 34]. Other studies have shown that inhibiting Transforming Growth Factor-*β*(TGF-*β*) signaling in thymic epithelial cells slows thymic involution [35] demonstrating that not only elevated Treg numbers but also increased Treg functions are associated with thymic involution. Therefore, physiological or pathological thymic involution affects the development of different T lymphocyte subpopulations at different ages.

On the other hand, the thymus gland and its different cell subpopulations are seriously injured by diverse parasites, which can induce sparse cellular apoptosis, tissue damage, or thymus involution, affecting the development, proliferation, migration, and the export to the periphery of T lymphocytes, and depressing the cellular immune response. In addition, parasites change the thymic microenvironment by increasing the intrathymic chemokine production and inflammatory cytokines production as well as extracellular matrix components [36].

For instance, in murine Chagas' disease model, the thymus is affected by severe thymocyte depletion mediated by corticosterone and excessive production of TNF-*α* associated with the export of immature CD4^+^CD8^+^ double-positive (DP) cells to the periphery [37]. Similar changes and thymic hypoplasia can be observed in experimental infections of mice with *Plasmodium berghei*. The parasites cause apoptosis of CD4^+^CD8^+^ thymocytes, exit of both these DP cells and DN thymocytes to mesenteric lymph nodes, as well as changes in the cortical and medullary limits of the thymus [38].

### 1.3. Care and Surveillance of Immature T Lymphocytes

Cells specialized in binding to other cells to assist them in their development are called “nurse cells.” Several types of cells in the body fit this definition. In particular, skeletal muscle cells in people infected with *Trichinella spiralis* are a classic example of nurse cells. Infected muscle cells retain the larvae of *Trichinella* in a cytoplasmic capsule, providing them with a protective envelope and nutrients from the host [39]. Thus, skeletal muscle “nurse cells” protect the *Trichinella* larvae from recognition, attack, or elimination by immune cells [40].

Some macrophages from the bone marrow also function as “nurse cells” secreting factors that stimulate the growth and development of just formed immature erythrocytes and absorbing their nuclei to enhance their oxygen transport [41]. Other cells with similar nursing functions are the bone marrow-derived fibroblastic stromal cells that infiltrate the synovial tissues of patients with rheumatoid arthritis (RA). Fibroblastic stromal cells contribute to inflammation and bone damage in RA by secreting cytokines and chemokines that trigger both the accumulation and activation of lymphocytes and monocytes in synovium [42]. Mesenchymal stromal cells and bone marrow-derived fibroblastic stromal cells in the synovium also promote antibody production and the survival of B cells, which contribute to synovium damage. Furthermore, Sertoli cells of the testes have been called “nurse cells” because they provide nutrients and growth factors to developing germ cells; therefore, impairment in Sertoli cell differentiation reduces spermatogenesis and testicular size [43].

The best known “nurse cells” are a subpopulation of epithelial cells of the thymus called thymic nurse cells that endocytose newly arrived immature T lymphocyte progenitors and sequester them in vacuoles named caveoles. Within these vesicles, the intercellular adhesion molecule 1 (ICAM-1) from TNCs interacts with lymphocyte function-associated antigen 1 (LFA-1) on thymocytes and activates signals necessary for lymphoid cell maturation [44]; other adhesion molecules enhance the selection of self-reactive T lymphocytes. Once these processes are completed, TNCs allow mature T lymphocytes to leave their cytoplasmic vacuoles and move into peripheral circulation. Defective thymocytes and autoreactive immature T lymphocytes that fail positive selection die by apoptosis inside nurse cells and are phagocytized by thymic resident macrophages [45].

Thus, the main function of thymic “nurse cells” seems to support the development and survival of healthy T cells and enhance the elimination of aberrant or damaged T cells. The protective role of TNC contrasts with the *Trichinella spiralis* nurse cells' deleterious functions, which promote invasive ability and survival of parasites and host's tissue damage.

### 1.4. Thymic Nurse Cells

Nurse cells of the thymus were discovered in mice thirty years ago [2], but later they had also been isolated from humans, rats, pigs, fish, ewes, frogs and chickens [46]. Nurse cells in the thymus are epithelial cells that temporally bind and internalize immature T lymphocyte progenitors in specialized vesicles [47] to help in their development, maturation, and selection [48]. Although thymic nurse cells are not easily studied *in vitro*, nurse cell lines are available by transforming nurse cells with the SV40 virus [49].

Thymic nurse cells are the major epithelial component in the thymus microenvironment and one of the major cell populations involved in monitoring the immature T lymphocytes' access to antigens that can stimulate or suppress their functions. However, the main function of TNCs is to participate in the positive and negative selection of immature T lymphocytes to tolerate self-antigens and eliminate foreign antigens [50]. Furthermore, the interaction between TNCs and immature T lymphocytes is necessary for their viability during their triple positive stage of intrathymic development [51].

Thymic nurse cells bind and internalize 50–200 immature TCR^−^CD4^−^CD8^−^ thymocytes in specialized cytoplasmic vesicles called caveoles, which are formed from the invaginations of the TNC plasma membrane [52]. A fine and detailed description of the internalization process of immature thymocytes into TNCs had been shown in Hendrix et al. [51].

Nurse cells of the thymus were initially described as multicellular complexes expressing cytokeratin 5 (K5) and/or cytokeratin 8 (K8) in their cytoskeleton [14] with a different location in the thymus. TNCs can express lysosomal-specific molecules and different proteases involved in the peptide/major histocompatibility complex (MHC) molecules—MHC class I and MHC class II—presentation. In human TNCs, laminin 211 has been detected [53], which is essential for the binding of these epithelial cells to immature T lymphocytes and in releasing T lymphocytes from the TNC vacuoles. Some TNCs also express the epithelial stem cell phenotype-associated transcription factor Trp-63 [51] and are K5+K8+ cells, suggesting that TNCs also possess different stages of development.

TNCs also express gap junctions formed by connexin 43 [54] and several P2Z purinergic receptors [55]. Both proteins are necessary in the communication of TNCs with each other, and in propagating calcium waves between neighboring nurse cells. TNCs secrete the hormone thymulin, which is necessary for the production of the Th1 cytokines interleukin-2 (IL-2) and interferon-gamma (INF-*γ*) in the thymus; they can also secrete IL-18, which helps in the development of fetal thymic CD11b^+^ dendritic cells and is also necessary for the induction of self-tolerance [56].

Furthermore, when TNCs are seeded and cultured on microplates, they produce the E_2_ and I_2_ prostaglandins [57] suggesting that TNCs may provide all these and other messages to immature T lymphocytes at specific developmental stages. Changes in the production of these endogenous and exogenous molecules can affect the balance on the events that modulates thymic microenvironment in which the development of T lymphocytes occurs.

On the other hand, TNCs are controlled by ligands and receptors from the endocrine and the nervous systems, since thymic epithelial cells express receptors for diverse hormones and neurotransmitters. Thymic nurse cells produce somatostatin, serotonin, gastrin [58], and the growth hormone [59] whose expression modulates the proliferation of T lymphocytes and thymic nurse cells and stimulates the secretion of thymic hormones, cytokines, chemokines, and extracellular matrix proteins in the thymic microenvironment, thus increasing thymocyte traffic inside and outside the thymus.

Nurse cells of the thymus also express some components of the cholinergic system [60] and acetylcholine. *In vitro* studies, using rat thymic epithelial cells [61] have demonstrated that adenosine triphosphate or noradrenaline induces the release of IL-6, a cytokine involved in thymocyte proliferation and differentiation. Besides, primary cultures of human and murine thymic epithelial cells produce thymulin [62] in response to beta-endorphin and leu-enkephalin added to the culture.

### 1.5. Distribution of Nurse Cells in the Thymus

In the thymus gland, nurse cells are distributed in both the cortical and the medullary zones of thymus lobules. In accordance with their location in the thymus, these populations are known as cortical thymic nurse cells (cTNCs) and medullary thymic nurse cells (mTNCs), respectively. These two TNC types share a common embryonic origin, but they exert different functions [63]. Some nurse cells are found within the corticomedullary interphase and express both K5 and K8, however.

cTNCs located in the subcapsular region of the thymus contain cytokeratin 8 in their cytoskeleton and engulf viable, immature, double negative (CD4^−^CD8^−^) thymocytes within TNC vacuoles. These cortex thymic nurse cells contain up to 200 immature thymocytes [64] inside caveoles. The inner membrane of caveoles expresses adhesion molecules called caveolins, intercellular adhesion molecule 1 (ICAM-1) and MHC class I and MHC class II molecules, by which cTNCs actively participate in the positive and negative selection of thymocytes [65]. Nurse cells of the thymus cortex also express several proteases such as cathepsin L, thymus-specific serine protease (TSSP) and the multicatalytic protease complex located in thymoproteosomes; all of them are involved in the positive selection of T lymphocytes after the cTNCs display unique self-antigens loaded onto MHC class I molecules [66].

Within cTNCs caveoles, the immature double-negative T lymphocytes transiently express CD25, the *α* chain of the IL-2 receptor [67] and begin to express CD4^+^ and CD8^+^ at low levels. Upon the induction of IL-7 and delta-Notch ligands, the DN T lymphocytes rearrange first the *β* chain and then the *α* chain of their TCR genes. Afterwards, they proliferate and maturate to the *αβ*TCR^high^ double-positive CD4^+^CD8^+^ stage [66] via an intermediate CD4^−^CD8^+^ single-positive, semimature stage. Thus, results suggest that cTNCs are involved in *αβ*T cell receptor-mediated positive selection.

In opposition to cortex thymic nurse cells, medullary thymic nurse cells contain semimature T lymphocytes within their caveoles, whose internal membrane surface expresses cytokeratin 5 (K5), and tissue-specific and tissue-restricted peptides. Peripheral self-antigens gain access to the thymus by both the blood supply and by differentiated dendritic cells that migrate from the periphery to the thymus [68]. Medullary TNCs also express the tissue-restricted antigens, insulin and thyroglobulin, the XC-chemokine ligand 1(XCL1), and the CCL19 and CCL21 chemokines to attract dendritic cells and positive selected thymocytes from the thymic cortex, respectively [69, 70]. The expression of these self-antigens in TNC is partially regulated by the transcription factor autoimmune regulator (AIRE), which also regulates tolerance in periphery [71].

Intravacuolar interactions of positively selected immature T lymphocytes with all these tissue-restricted peptides are decisive steps for establishing self-tolerance. For this reason, the main functions of medullary thymic epithelial nurse cells are associated with the negative selection of autoreactive T lymphocytes and the establishment of a self-tolerant T cell repertory.

In addition, medullary thymic epithelial cells that express tissue-specific self-antigens indirectly participate in the generation of natural Tregs [72] with the help of dendritic cells. Simultaneously, mTNCs induce the development of T-regulatory cells to compensate the incomplete presentation of self-antigens in the thymus [73].

### 1.6. The Positive and Negative Selection of Thymocytes

The selection of thymocytes in the thymus is an MHC-TCR-restricted process associated with the recognition of self-antigens. This process is necessary to complete the maturation of T lymphocytes (positive selection) or to eliminate autoreactive T lymphocytes by apoptosis (negative selection). Interactions between cortical thymic nurse cells and semimature T lymphocytes through the affinities of the MHC/peptide-TCR*αβ* define the further fate of lymphoid cells. Strong affinities with self-antigens trigger the deletion of immature T lymphocytes, minimal affinities lead to death by neglect and intermediate affinities promote positive selection by survival and differentiation of CD4^+^CD8^+^ double-positive thymocytes to either the CD4^+^CD8^−^ or CD4^−^CD8^+^ single-positive linage [74].

In the thymic cortex, semimature T lymphocytes use their new TCRs to recognize self- and non-self antigens associated with MHC molecules on TNCs or dendritic interdigitating cells. CD4^+^CD8^+^ double-positive semimature T lymphocytes inside cTNC that recognize self-antigens with low avidity through their *αβ*T cell receptor (TCR) are positively selected [66], as well as those DP semimature T lymphocytes that produce low intensity activating signals through their TCR [10]. Thus, the main function of cortex TNC appears to support the positive selection of CD4^+^ or CD8^+^ T cells [75].

Positive selected CD4^+^ or CD8^+^ T lymphocytes are viable semimature cells transitorily contained in TNC vacuoles, where they express the Qa2^low^CD62L^low^HSA^high^CD69^high^CD24^high^ phenotype, which is susceptible to apoptosis [76]. As T lymphocytes differentiate and express the Qa2^high^CD62L^high^HSA^low^CD69^low^CD24^low^ new phenotype, they are transformed in mature cells refractory to apoptosis and are released from the thymus to start their own immunological functions [10]. In addition, when immature T lymphocytes from TNCs have been cultured, they show a decrease in their Bcl-2 expression [77]. This result suggests that they may be less susceptible to apoptosis because of the protection conferred by TNCs [78].

In the neonatal period, T lymphocytes positively selected (maybe 2%–5%) receive a survival signal, express receptors (CCR7 and CCR9) for CC chemokines and move into the thymic medulla [79], although the CCR7 is not necessary for their emigration from the thymus in adulthood. During their migration into thymus medulla, immature T lymphocytes acquire a single-positive (SP) phenotype either the CD4^−^CD8^+^ or the CD4^+^CD8^−^, both of which are dependent on the recognition of MHC class I or MHC class II molecules by their TCRs [80]. Semimature double-positive cells are developed by stronger or sustained signals from TCRs through the small GTPase RasGRP1, and the kinase ERK yielding CD4^+^ SP thymocytes; meanwhile weaker or transient signals on TCRs produce CD8^+^ SP thymocytes.

When high affinity TCRs of developing T lymphocytes recognize self-antigens/self-MHC complex in TNC vacuoles or on sparse thymic dendritic cell surface, they do not leave the TNC caveolae [45] and die by apoptosis inside cortical thymic nurse cells or survive and then leave the thymus but into a state of unresponsiveness or anergy.

In this way, immature T cells make their first immunological synapses with MHC molecules when they are inside TNC caveoles. The recognition of TNC-derived MHC molecules is a critical event for both the positive and negative selection of immature T lymphocytes upon expression of their *αβ*TCR [81]. Antibodies specific for either MHC class I or MHC class II molecules reduce the release of viable T lymphocytes from TNCs to the culture medium [78], thus suggesting that TNCs rescue immature T lymphocytes from apoptosis through the TCR-MHC interaction.

### 1.7. The Kiss of Death

Although TNCs can protect some immature T lymphocytes from apoptosis [77], the lymphoid cells confined within TNC caveoles are killed in most cases. The synapse between the internal membrane of the TNC caveolae and the TCR on the membrane surface of immature T lymphocytes acts as a kiss of death for autoreactive cells. After the high-affinity TCR-mediated recognition of self-antigens expressed on TNC caveoles, autoreactive thymocytes remain arrested within the nurse cells and are eliminated there by apoptosis. The event is called negative selection.

Thymic epithelial cell-mediated apoptosis needs cell priming, and 98% of the CD4^+^CD8^+^ double-positive immature T lymphocytes undergo apoptosis since they are more sensitive to the BAD, PUMA, and HRK-1 apoptotic sensitizer proteins [82] than are single-positive CD4^+^ or CD8^+^ cells. The epithelial thymic cell-mediated apoptosis of double-positive immature T lymphocytes is also induced by nitric oxide [83], which synergizes with glucocorticoids and activates cathepsin B and caspase-3, -8, and -9 [84].

On the other hand, adenosine also induces apoptosis into the thymus. *In vitro* studies have demonstrated that the apoptosis of mouse autoreactive immature T lymphocytes requires both high doses of adenosine produced by macrophages and adenosine A2 receptors expressed in immature lymphoid cells. The activation of A2 receptors by adenosine induces a Bcl-2-mediated apoptotic process that needs the proapoptotic protein Bim. In humans, thymocyte apoptosis also involves the Ca2^+^-dependent induction of the transcription factor Nur77, a member of the steroid/thyroid hormone receptor superfamily [85] that binds to the promoters of the Fas ligand, TNF-related apoptosis-inducing ligand, and NDG-1 and -2 apoptosis-inducer proteins.

Once apoptosis is induced in autoreactive immature T lymphocytes, the epithelial TNCs recognize them via the scavenger receptor B1, a high-density lipoprotein receptor and a phosphatidylserine receptor and then the thymocytes undergoing apoptosis are retained within TNC caveoles. There, apoptotic cell residues are eliminated by lysosomal enzymes from TNCs [86], resident thymic cortex macrophages, or peripherally-recruited macrophages that arrive at the thymus when thymocyte apoptosis is increased [87]. Electron microscopy images from the cortex of the human thymus have shown that macrophages surround TNCs [88]. Macrophages have also been revealed within the vacuoles of TNCs [89], where macrophages move in and out rapidly. In this way, the physiological elimination of autoreactive thymocytes is performed and prevents the further development of autoimmune diseases.

The fatal kiss between the thymic nurse cells and their protected immature lymphocytes is a physiological event that has critical consequences for the development of self-tolerance in the immune system [77]. Self-antigens presented by mTNCs are essential for the negative selection of dangerous autoreactive maturing lymphocytes that must die in the thymic nursery [48]. Immune tolerance for self-antigens can be disrupted by defective elimination of autoreactive thymocytes that have a deficient expression of the proapoptotic protein Bim [90], mutations on the tyrosine kinase ZAP-70 signaling [91] or the depressed expression of the Mer tyrosine kinase [92]. These disorders have been associated with the deficient elimination of autoreactive cells by negative selection [47] resulting in the induction of autoimmune diseases.

The negative selection of autoreactive lymphocytes does fail however, since the deletion of these defective cells is incomplete, and some autoreactive T lymphocytes evade the kiss of death in the TNC microenvironment [93] and move into the peripheral blood circulation. In general, these autoreactive cells do not induce autoimmune diseases cause they are controlled by T regulatory (Treg) cells, which suppress peripheral T lymphocyte-mediated responses against self-antigens [94]. Nevertheless, the loss of the homeostasis between TNC and Treg cell functions can result in the excessive output of autoreactive T lymphocytes or defects in the suppression of T cells reactivity.

### 1.8. The Regulatory T Cells

Once T lymphocytes mature and leave the thymus, their surveillance and defensive functions begin to be controlled by other accessory cells called regulatory T (Treg) lymphocytes. The natural Treg cells are thymus-derived T lymphocytes, which modulate many aspects of the normal immune responses, suppressing inflammation, hypersensitivity, or autoimmune mechanisms. However, not all Treg cells develop in the thymus gland [95].

Moreover, not all T regulatory cells exert a suppressor role since Tregs subpopulations with a proinflammatory function have been reported [96]. Tregs are specifically activated by antigens although their effector function is antigen nonspecific [97]. Besides, the self or non-self antigen binding specificity of the Treg lymphocyte through their TCR does not influence their selective process within the thymus gland when immature Treg cells are under the influence of cytokines, hormones, and neurotransmitters. In addition, Treg lymphocytes exert their suppressive functions on other immune cells [98] by releasing cytokines, through cell-cell contact, or by inducing cytotoxicity or anergy in antigen presenting cells [99].

**Figure 1:**
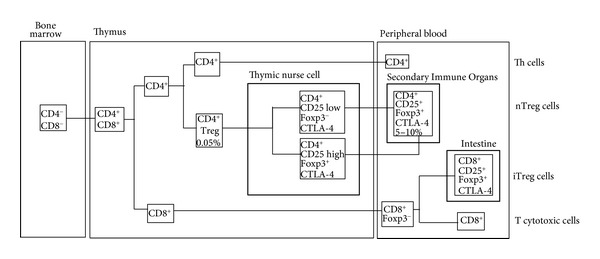
The intra- and extrathymic origin of T regulatory (Treg) lymphocytes. The bone marrow-derived pre-T lymphocytes arrive at the thymus microenvironment as double negative CD4^−^CD8^−^ cells, which are engulfed by thymic nurse cells, where they mature to CD4^+^ or CD8^+^ lymphocytes or are negatively selected. A reduced proportion (0.05%) of the CD4^+^ lymphocytes become regulatory cells by expressing CD25, Foxp3, CTLA-4, and other molecules. When this subpopulation of natural T regulatory (nTreg) lymphocytes is mature, they are released from the medullary thymic nurse cells, leave the thymus, and go into the blood and peripheral lymphoid organs where they release IL-10 and TGF-*β* suppressor cytokines that downmodulate the functions of other cells from the immune system. A different subpopulation of T regulatory cells can be experimentally induced from CD8^+^ cytotoxic lymphocytes located outside the thymus. These lymphocytes exert an *in vitro* suppressor activity through IL-10, IL-4, and TGF-*β*; they are called inducible T regulatory (iTreg) lymphocytes and have been found as infiltrating cells with an effective antitumor activity.

Treg cells developed in the thymus are referred to as “natural” Treg (nTreg) cells, whereas “adaptive” or “induced” Treg (iTreg) cells are either naïve peripheral Foxp3^−^CD4^+^ T lymphocytes or Foxp3^−^CD8^+^ T lymphocytes, which develop in the periphery upon subimmunogenic antigen presentation during chronic inflammation or normal homeostasis of the gut after T lymphocytes recognize foreign antigens [100, 101]. The origen and location of Treg cells in the body is depicted in Figure 1.

Natural Treg cells are long-lived cells that comprise a very small subpopulation of thymus-derived CD4^+^ T cells (5–10%). They are produced as a result of the high-affinity TCR-self peptide : MHC class II molecule interactions between maturing thymocytes and TNCs requiring also of external stimuli such as IL-2, CD80 and CD86. Natural Tregs constitutively express the alpha chain of the IL-2 receptor (CD25), need TGF-*β* and IL-2 for their maduration and are neither phenotypic nor functionally homogeneous [102]. Natural human Tregs (70%) express the Helios transcription factor, which regulates both Foxp3 expression and regulatory T cell activity [103], and possess the Foxp3^+^Helios^+^ phenotype. Notwithstanding, Foxp3^+^Helios^−^ Tregs can be expanded *in vitro* into Foxp3^+^Helios^+^ T regulatory cells by adding a DNA oligonucleotide [87].

On the other hand, induced Tregs may play an important role in the immune tolerance of foreign antigens such as those derived from commensal bacteria in the intestine [104], and their functions are essential and complementary to the regulatory function of nTregs. Natural or induced T regulatory cells have different origins and specificities and exert their functions through distinct mechanisms. An excellent review regarding the role of self-reactivity as the decisive factor in Treg development in the thymus has just been published [105].

Other Treg subsets have been proposed, such as the Type 1 regulatory T cells (Tr1) and T-helper-3 (Th3) lymphocytes. The former are T cells, which produce TGF-*β* and IL-10 upon antigen exposure and specific tolerogenic conditions, and the latter produces TGF-*β* upon intestinal tolerance [106]. Accordingly, Tregs are stimulated by different agents and possess different properties.

### 1.9. The Heterogeneity of Treg Cells

The Treg subpopulation contains suppressor and effector/memory cells that also control tumors and pathogens [101]. The same as all T cells, natural T regulatory cells are long-lived and migrate from the thymus into the periphery and secondary lymph tissues, where they also balance self-tolerance and autoimmunity. Previously, Coutinho et al. [107] have proposed that Tregs are selected in the thymus upon high-affinity recognition of self-ligands in cortex thymic epithelial stromal cells.

All Tregs acquire their specific phenotype and functions when they upregulate the expression of Foxp3, which expression requires Signal Transducer and Activator of Transcription-5 (STAT-5) activation driven by IL-2 [108]. However, it is worth keeping in mind that epithelial and other subtype of cells such as cancer cells can also express the Foxp3 protein [109]. Moreover, Treg cells are modulated by cytokines released by cells that express Toll-like receptors such as TLR-2 and TLR-4, as has been reported in patients with atopic dermatitis [110] and hepatoma cell lines [111], in a context that has not yet been fully explored.

In the thymus, Tregs are thymocytes that develop from Foxp3^−^CD4^+^CD25^+^ cells and appear more frequently during the transition of the late CD4^+^CD8^+^ DP stage to the final CD4^+^CD8^−^ or CD4^−^CD8^+^ SP stage [112]. T regulatory cells also express the CD45RB^high^ or CD45RB^low^, CD38^+^ or CD38^−^, CD69^+^ or CD69^−^, and CD62L^high^ or CD62^low^ membrane markers [113]. However, there are CD4^+^CD25^−^ T cells possessing regulatory functions. Other surface markers expressed in Tregs are CD127^low^, HLA-DR, CD103, CD39, Neuropilin-1 (Nrp-1), and Tumor Necrosis Factor receptor family-related members such as the Glucocorticoid-induced TNF receptor (GITR/TNFRSF18), OX-40, and CD137.

The activated CD8^+^CD25^+^ natural Treg lymphocytes are thymus-derived cells that share phenotypic and functional characteristics of the CD4^+^ Tregs, since they also express the cytotoxic T lymphocyte-associated antigen 4 (CTLA-4), the Glucocorticoid-induced Tumor Necrosis Factor receptor (GIRT), and the Transforming Growth Factor-*β*1 [98, 114]. They comprise less than 1% of CD8^+^ lymphocytes.

In addition, diverse CD8^+^ Tregs subtypes exists, which are naturally produced or induced with cytokines such as IL-4, IL-10, Granulocyte-Macrophage Colony-Stimulating Factor (GM-CSF), IL-2, INF-*γ* and TGF-*β*, viral antigens, xenogeneic antigen presenting cells, allogeneic stimulation, non-antigen specific stimulation, cocultures with monocytes, cocultures with LPS-stimulated dendritic cells (DCs), and plasmacytoid DC from tumor ascites [98] among others.

Surprisingly, a fraction (10–15%) of CD25^+^ natural Treg cells never express or lose the transcription factor Foxp3^+^ when they proliferate in a T cell deficient environment [108]. Some of these Treg-derived Foxp3^−^ T cells exert an effector T helper function while others maintain their ability of expressing Foxp3 upon activation [115], showing that T regulatory cells possess plasticity. The Foxp3^+^ T regulatory cells can also functionally differentiate to control the T_H_1, T_H_2, and T_H_17 cell response [116] by changing the expression of the T_H_ lineage-specific transcription factors T-bet, Interferon Regulatory Factor 4 (IRF-4), and Orphan Nuclear Receptor *γ*t (ROR*γ*t), respectively.

Different cells and molecules are involved in the Treg development in the thymus. Accordingly, T cell expression and signaling, the expression and signaling of CD11a/CD18, CD28, and CD40L on thymocytes, ICAM-1 and CD80, CD86, and CD40 expressed on thymic stromal cells [3] control Tregs development and the selection of their heterogeneous subpopulations [105]. The expression of MHC class II molecules on medullary TEC also promotes Treg development [75]. Tregs develop if the MHC-TCR affinity is high [111], although the affinity for the development of the CD4^+^CD25^+^ thymocytes is different from that of CD4^+^CD25^−^ thymocytes. Dendritic cells induce the differentiation of T regulatory cells [117]. TGF-*β* is needed for the Foxp3 expression in Treg cells [108], and glucocorticoids stimulate Treg activity [118]. Tregs need B-lymphocytes to survive and proliferate on the periphery [113]. In addition, IL-2, IL-7, and IL-15 are required for the peripheral maintenance of Tregs, and they are also probably needed for the survival of immature Tregs in the thymus medulla [112]. 

### 1.10. Blood Levels and Functions of the Treg Cells

**Figure 2:**
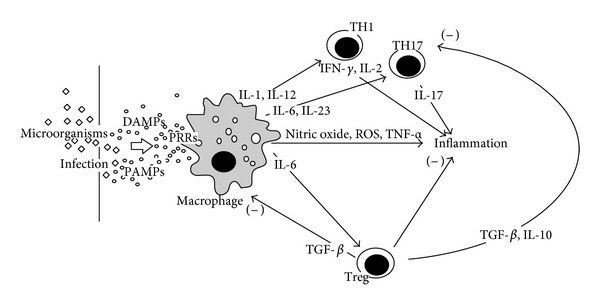
A defensive inflammatory response starts after the pattern recognition receptors (PRRs) of macrophages and dendritic cells are stimulated by both pathogen-associated molecular patterns (PAMPs) released by microorganisms and damage-associated molecular patterns (DAMPs) from injured tissues. As a consequence, diverse signaling pathways increase both the production of proinflammatory cytokines and the release of free radicals during the cellular respiratory burst. The evolution of the inflammatory response is modulated by various subpopulations of cells including T lymphocytes. The proinflammatory T lymphocytes (Th1 and Th17) mainly release IL-2, IL-17, and IFN-*γ*, and the antiinflammatory lymphocytes (Treg) release TGF-*β* and IL-10. The effective modulatory work of Treg cells gradually slows down the progression of the inflammatory responses and reduces any possible risk of autoimmunity, allergies, or other chronic diseases.

Once Treg cells have been developed, their numbers can be increased or decreased by diverse diseases or physiological conditions, which can influence their suppressor functions according to the quality of the required immune response. In addition, the number and functions of Tregs cells are necessary in controlling autoimmunity since depletion of CD4^+^CD25^+^ T cells in mice, mutations in the Foxp3 gene, or environmental agents affecting Treg cells cause or predispose to autoimmunity [116] because the lack in balancing the activity of self-reactive T cells produced in the thymus. Thus, CD4^+^ Tregs or CD8^+^ Tregs appear impaired in number and/or function in diverse autoimmune diseases [98] such as lupus erythematous, autoimmune diabetes, rheumatoid arthritis, myasthenia gravis, multiple sclerosis, allergy, inflammatory bowel disease, hepatitis C, herpes simplex, HIV infections, and cancer. However, Treg lymphocytes are frequently expanded in physiological conditions such as aging [119] and pregnancy [120]. An overview regarding the role of the Treg cells in controling infection, inflammation and the function of other T lymphocytes are depicted in Figure 2.

Experimental models of parasitic infection with *Strongyloides ratti* [121] and *Trichuris muris* [122] increase the number of Foxp3^+^ Treg lymphocytes suppressing the protective immune response and probably reducing the parasite-induced damage in the host. Similar results have been observed during experimental acute or chronic malaria infection by *Plasmodium chabaudi* in C57BL/6 mice [123] and in filaria-infected non-obese diabetic mice [124].

Other different studies have shown that there is also an inverse relationship between the peripheral nonphysiological increase in Treg numbers and thymic involution [33]. The numbers of Treg cells in the blood increases as a consequence of thymic involution and lymphopenia, as can be observed in children with the Down syndrome [125]. In opposition, low numbers of circulating Treg cells have been associated with the onset of allergic and autoimmune responses [126]. Other authors have reported that the proportion of Treg cells increases in the spleen and lymph nodes of mice with experimentally induced arthritis [127].

Although deficient T lymphocyte-mediated immune responses are usually associated with elevated amounts of Treg cells in the blood, the increased production of proinflammatory cytokines such as IL-17 and IL-18 and the elevated number of Th17 lymphocytes are frequently associated to decreased numbers of Treg lymphocytes in the blood and lymphoid organs [128].

In support of the role for Treg cells in the prevention of immunopathology, it has been reported that the experimental depletion of Foxp3^+^ Treg cells reduces the control of the inflammatory immune responses, increases the frequency of autoimmune reactions due to TCR-mediated self-antigen recognition and enhances the development of a scurfy-like disease in mice, which die at 3-4 weeks of age [129]. In contrast, Foxp3^+^ Treg cells from mice deficient in CD5, a negative regulator of TCR signaling increases the suppressive activity of their Treg cells [97].

Furthermore, the presence of cellular markers of Treg cells in the microenvironment of thymic nurse cells [71] suggests a relationship between autoimmunity and disorders in the intra-TNC maturation of Treg cells. Incomplete Treg cell development in TNCs can result in defective Treg cell suppressor activity and consequently in the development of autoimmune reactions or diseases. For example, nonobese diabetic (NOD) mice are diabetic because of a defect in their antigen presenting cells to activate Treg cells. NOD mice also have Treg cells, which are defective in their regulatory function and possess lower percentages of CD4^+^CD25^+^ T regulatory cells than NOD mice that never develop diabetes [130].

The extremely complex relationship between Treg cells and autoimmunity is a function not only of the defective suppressor activities of peripheral Treg cells, however. Neurotransmitters and sex hormones have recently been added to the long list of modulators of the Treg cell function. Thus, *in vitro* experiments show that pituitary adenylyl cyclase-activating polypeptide (PACAP) [131], vasoactive intestinal peptide [132], and nicotine [133] increase the suppressor activity of Treg cells. Furthermore, studies of cultured fluorescence-activated-sorted Tregs from pregnant women and peripheral blood mononuclear cells from nonpregnant women have revealed that progesterone and 17 *β*-estradiol especially decrease the expression of Foxp3 in CD4^dim⁡^CD25^high^ Treg cells [134] as well as their cell numbers, although these Tregs cells maintain their regulatory function. In contrast, other authors have shown that oestrogen enhances the frequency of Treg cells and reduce the production of IL-17 in a mouse model of multiple sclerosis [135].

The suppressive control of T lymphocyte responses is a complex event in which the major inductors are Treg cells modulated by numerous factors coming from the immune system or not.

### 1.11. Regulating the Regulators

Since immune system responses are regulated by Treg cells and MHC class II-expressing nurse epithelial cells from the thymus regulate the development of Treg cells [75, 136], a question arises: what regulates both the development and the functions of thymic nurse cells? To answer this question, most researchers have focused their attention outside the thymus gland, specifically on the endocrine [137] and the nervous [138, 139] systems.

Understanding the interactions between the nervous and immune systems has considerably increased [140] in the last three decades, and a great deal of studies have shown that these interactions are possible because immune and nervous cells share receptors for several neurotransmitters and cytokines [139]. Moreover, the innervation of the thymus gland, bone marrow and all secondary organs of the immune system by the autonomous nervous system are essential for the functioning of the immune system [140].

Accordingly, the connective tissue of the thymus contains non-myelinated nerves forming a lattice over their surface [141]. These nerves contain calcitonin gene-related peptide (CGRP), noradrenaline, substance P (SP), vasointestinal peptide (VIP), and neuropeptide Y (NPY) [142–148]; other nerves have acetylcholinesterase, which can affect the thymocyte development. The direct effect of GABA, Histamine, NPY, SP, VIP, and CGRP on the proliferation of rat thymic epithelial cells was reported years ago [149]. The P2Z receptors, a kind of purinergic receptor, were later demonstrated to exist in thymic epithelial nurse cells [150]. In addition, catecholamines (adrenaline, noradrenaline, and dopamine) from the sympathetic nervous system have a main role in controlling lymphocyte development and immunomodulation [151].

Since the thymus is innervated by the sympathetic nervous system through norepinephrine [141], sympathectomy of adult rats results in a reduction of thymus weight, decreased intrathymic cellularity and increased T cell apoptosis [143] by affecting noradrenergic, vasointestinal peptide, acetylcholine and CGRP nervous fibers in the thymus. In contrast, other authors [144] have shown that the peripheral administration of 6-hydroxydopamine (6-OHDA) increased the numbers of CD4^+^Foxp3^+^ Tregs in the spleen and lymph nodes in a TGF-*β*-dependent manner without affecting their regulatory function or the frequency of all CD4^+^ and CD8^+^ T cells.

On the other hand, *in vitro* experiments have confirmed that cultured thymic epithelial cells express both the *α*, *β*, and *ε* subunits of the acetylcholine receptor similar to those shown in muscle cells [20] and functional *β*1 and *β*2 mRNA to express adrenoreceptors, activated by adrenaline and noradrenaline [143]. Besides, the adrenergic agonist carbachol inhibits apoptosis of DP thymocytes by TECs [146]. Furthermore, immunohistochemistry assays have shown that epithelial cells of the rat thymus are close to catecholaminergic nerves controlled by dopamine [147].

A possible role for acetylcholine has been also proposed in the mutual interplay between immature lymphocytes and thymic epithelial cells because the TE750 thymic epithelial cell line and primary thymic epithelial cell cultures have shown *in vitro* the expression of acetylcholine, the *α*3, *α*5, and *β*4 mRNA subunits of one cholinergic receptor and choline acetyltransferase [60]. Outside the thymus gland, the histamine released by bone marrow-derived mast cells inhibits the *in vitro* suppressor function of Treg lymphocytes and downregulate the expression of CD25 and Foxp3 markers of CD4^+^CD25^+^ cells [152] via the histamine 1 receptor expressed by these cells.

Other studies have revealed that the levels of the inhibitory neurotransmitter gamma-aminobutyric acid (GABA) are elevated in the thymus during the immune response [153] as compared with other glands. Epithelial cells in thymic medulla expresses the glutamate decarboxylase-67 isoform, which predominantly synthesizes GABA in the central nervous system [154], and the thymus also expresses high GABA-transaminase activity after IL-1 stimulation [155]. Nevertheless, the expression of components of the GABAergic system or GABA receptor subunits has not been demonstrated in thymic epithelial cells. We have found evidence for a GABAergic system in mouse peritoneal macrophages [156], but the presence of a similar system in intrathymic macrophages has not been addressed.

Regarding the role of GABA on the thymus and lymphocytes, Tyurenkov et al. [157] demonstrated that baclofen, a GABA-B receptor agonist, restores thymus weight and thymocyte numbers after experimental immunosuppression. Notwithstanding its inhibitory effect on the cells from the nervous system, GABA increases the proliferation of rat thymic epithelial cells cultured *in vitro* [149], whilst its oral administration inhibits both diabetes development in type 2 diabetic mice [158] and inflammation in fat diet-fed mice [159] by increasing the frequency of their Tregs cells. These two later *in vivo* results suggest that GABA administration may be a useful tool that enhance the effects of the conventional therapy for preventing type 1 diabetes and other T lymphocyte-mediate autoimmune diseases in mice, although the role of GABA in the TNC-Treg cells interaction remains unexplored.

Thymic nurse cells are the main engaged cells in the development and selection of immature T lymphocyte in the thymus. Although relationships between TNCs and the nervous system have been emerging in the last decades, their data are scanty. Even more limited are the studies regarding the relationships between neurotransmitters or neuropeptides and the density of their receptors expressed on immune cell subsets [160]. Further studies are needed to unveil the existing interactions between the nervous system and both TNC and Tregs cells completely. Understanding the mechanisms by which the vegetative nervous system regulate the TNC functions and development through neurotransmitters and neuropeptides may be helpful in controlling autoimmune diseases, transplants, inflammation, and allergy.
